# Diselenide-Bridged Doxorubicin Dimeric Prodrug: Synthesis and Redox-Triggered Drug Release

**DOI:** 10.3390/molecules29081709

**Published:** 2024-04-10

**Authors:** Yanru Hu, Peng Liu

**Affiliations:** State Key Laboratory of Applied Organic Chemistry, College of Chemistry and Chemical Engineering, Lanzhou University, Lanzhou 730000, China; huyr20@lzu.edu.cn

**Keywords:** tumor chemotherapy, dimeric prodrug, diselenide bond, redox triggered, combination of conjugations, doxorubicin

## Abstract

The diselenide bond has attracted intense interest in redox-responsive drug delivery systems (DDSs) in tumor chemotherapy, due to its higher sensitivity than the most investigated bond, namely the disulfide bond. Here, a diselenide-bridged doxorubicin dimeric prodrug (D-DOX_SeSe_) was designed by coupling two doxorubicin molecules with a diselenodiacetic acid (DSeDAA) molecule via α-amidation, as a redox-triggered drug self-delivery system (DSDS) for tumor-specific chemotherapy. The drug release profiles indicated that the D-DOX_SeSe_ could be cleaved to release the derivatives selenol (DOX-SeH) and seleninic acid (DOX-SeOOH) with the triggering of high GSH and H_2_O_2_, respectively, indicating the double-edged sword effect of the lower electronegativity of the selenide atom. The resultant solubility-controlled slow drug release performance makes it a promising candidate as a long-acting DSDS in future tumor chemotherapy. Moreover, the interaction between the conjugations in the design of self-immolation traceless linkers was also proposed for the first time as another key factor for a desired precise tumor-specific chemotherapy, besides the conjugations themselves.

## 1. Introduction

Although chemotherapy has been widely used in clinical tumor treatment, it is still severely toxic and has side effects for patients receiving anticancer treatment. Due to their nonspecificity, chemotherapeutic drugs exhibit similar cytotoxicity on both tumor cells and normal cells. Furthermore, the clinical practice of chemotherapeutic agents is restricted due to their poor aqueous solubility. Therefore, tumor-specific chemotherapy is desired with nanoscaled drug delivery systems (DDSs) via different targeting strategies to improve the antitumor efficacy and restrain any toxicity and side effects. Compared with passive targeting via an enhanced permeability and retention (EPR) effect [1] and active targeting via overexpressed receptors [2] on the tumor cells, smart targeting has been recognized as a promising approach as it involves both the tumor intracellular microenvironment-activated drug release, which is triggered by endogenous stimuli, such as higher glutathione (GSH) and reactive oxygen species (ROS) levels than normal cells, and the extracellular environment, as well as the specific enzymes in the tumor cells [3]. For example, in recent decades, the disulfide bond has been intensely investigated in redox-triggered DDSs for tumor-specific drug delivery with minimized premature drug leakage before reaching the tumor cells [4].

Recently, selenide-containing compounds have attracted more attention in tumor treatment, owing to their anticancer and/or chemopreventive activity [5,6]. Among them, the diselenide bond has become a research highlight in redox-responsive DDSs for tumor chemotherapy, because of its higher redox-responsive sensitivity than the most widely investigated disulfide bond [7,8,9]. To date, various redox-responsive drug carriers have been designed for noncovalent drug loading by crosslinking with diselenide-containing linkers. Besides the endogenous stimuli-responsive tumor-targeted drug release via the reduction-responsive de-crosslinking triggered by GSH and/or oxidation-responsive de-crosslinking triggered by ROS in the tumor intracellular microenvironment [10,11,12,13,14,15,16,17,18,19], it can also be cleaved with exogenous stimuli, such as X-ray [20,21,22], γ-ray [23] and near-infrared (NIR) laser [24,25], accelerating the drug release. Moreover, selenide-containing degraded products can disrupt intracellular redox homeostasis and amplify oxidative stress in cancer cells [26].

However, carrier-mediated DDSs usually possess lower drug content, besides having problems related to the toxicity and immunogenicity of the carriers. In particular, for DDSs using noncovalent drug loading, premature drug leakage is more significant, causing toxic side effects on normal tissues. Owing to the higher redox-responsive sensitivity, the premature drug leakage from diselenide-crosslinked DDSs, triggered by very low GSH levels in the blood (~10 μM), is more severe than disulfide-crosslinked systems. Such problems are also present in prodrugs, in which chemotherapeutic agents are conjugated onto carriers via the diselenide bond [27], as well as their low drug content and the cytotoxicity and immunogenicity of the carriers.

To solve such problems, dimeric prodrugs have been proposed as carrier-free drug self-delivery systems (DSDSs) with a high drug content, almost to the level of pure drugs [28]. Thus far, the diselenide-bridged dimeric prodrugs that have been reported involve linking two drug molecules with a diselenide-containing linker via α (β or γ) esterification on the hydroxyl group of the drugs [29,30,31]. Although the parent drugs could be released by redox cleavage and hydrolysis, a distinct premature drug leakage was found even in the blank-releasing media without any stimulus (~8%) due to the high redox responsiveness of the diselenide bond [29]. Such DSDSs would cause more severe drug mis-release in normal cells with higher GSH levels (~2 mM). These results indicated that the desired precise tumor-specific drug release could not be achieved with the DSDSs controlled by the combination of α (β or γ)-ester and diselenide bonds.

**Scheme 1:**
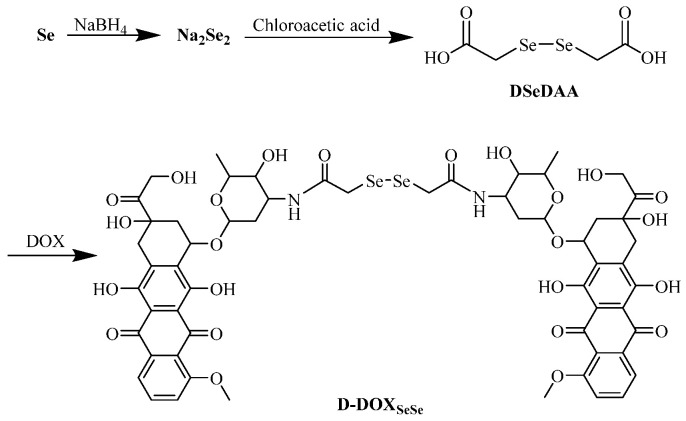
Synthesis of the diselenide-bridged doxorubicin dimeric prodrug (D-DOX_SeSe_).

Here, a diselenide-bridged doxorubicin dimeric prodrug (D-DOX_SeSe_) was designed by coupling two doxorubicin (DOX) molecules with a diselenodiacetic acid (DSeDAA) molecule via α-amide groups (Scheme 1). Triggered by high levels of GSH or H_2_O_2_, the derivatives of the parent drug DOX, selenol (DOX-SeH), and seleninic acid (DOX-SeOOH) were released. Thus, a solubility-controlled slow drug release was achieved without drug leakage and mis-release in the media with lower GSH or H_2_O_2_ levels. This meant that the amide linker could not be induced to hydrolyze by selenol or seleninic acid in the drug derivatives, different from those with the combination of α-ester and diselenide bonds [26], as well as the combination of α-amide and disulfide bonds [30]. The drug release behavior demonstrated the double-edged sword effect of the selenide atom with lower electronegativity than the sulfur atom. Additionally, we suggest that the interaction between conjugations be considered in the design of self-immolation traceless linkers as another key factor, besides the conjugations themselves. Such understanding is quite significant to the design of smarter prodrugs for tumor-specific on-demand drug release in future tumor chemotherapy.

## 2. Results

### 2.1. Synthesis and Characterization of Diselenide-Bridged Doxorubicin Dimeric Prodrug

**Figure 1:**
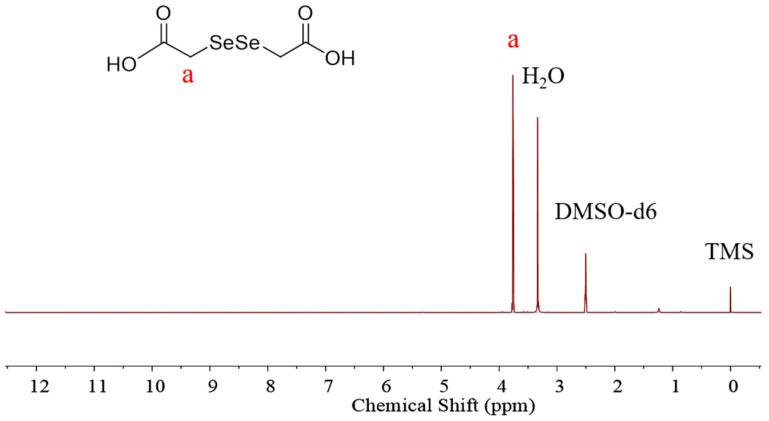
^1^H NMR spectrum of DSeDAA.

The diselenide-bridged doxorubicin dimeric prodrug (D-DOX_SeSe_) was developed by designing a combination of α-amide and diselenide bonds with DOX and DSeDAA (Scheme 1). DSeDAA was synthesized with selenide, sodium borohydride (NaBH_4_), and chloroacetic acid, according to previously reported work [31]. Due to the symmetrical structure, there was only one proton signal at δ = 3.76 ppm (Figure 1).

Then, the diselenide-bridged doxorubicin dimeric prodrug (D-DOX_SeSe_) was synthesized by coupling two DOX molecules with a DSeDAA molecule. Owing to the higher activity of the amino group than the hydroxyl groups, the amino group on DOX was amidated with DSeDAA to form the proposed diselenide-bridged doxorubicin dimeric prodrug via a combination of β-amide and diselenide bonds. After purification via dialysis with water to remove the residual reactants, D-DOX_SeSe_ was obtained, and its purity was revealed with thin-layer chromatography (TLC) on a silica gel plate. The C, H, and N element contents were determined as 52.52%, 4.52%, and 2.10%, respectively, very near to the theoretical values.

**Figure 2:**
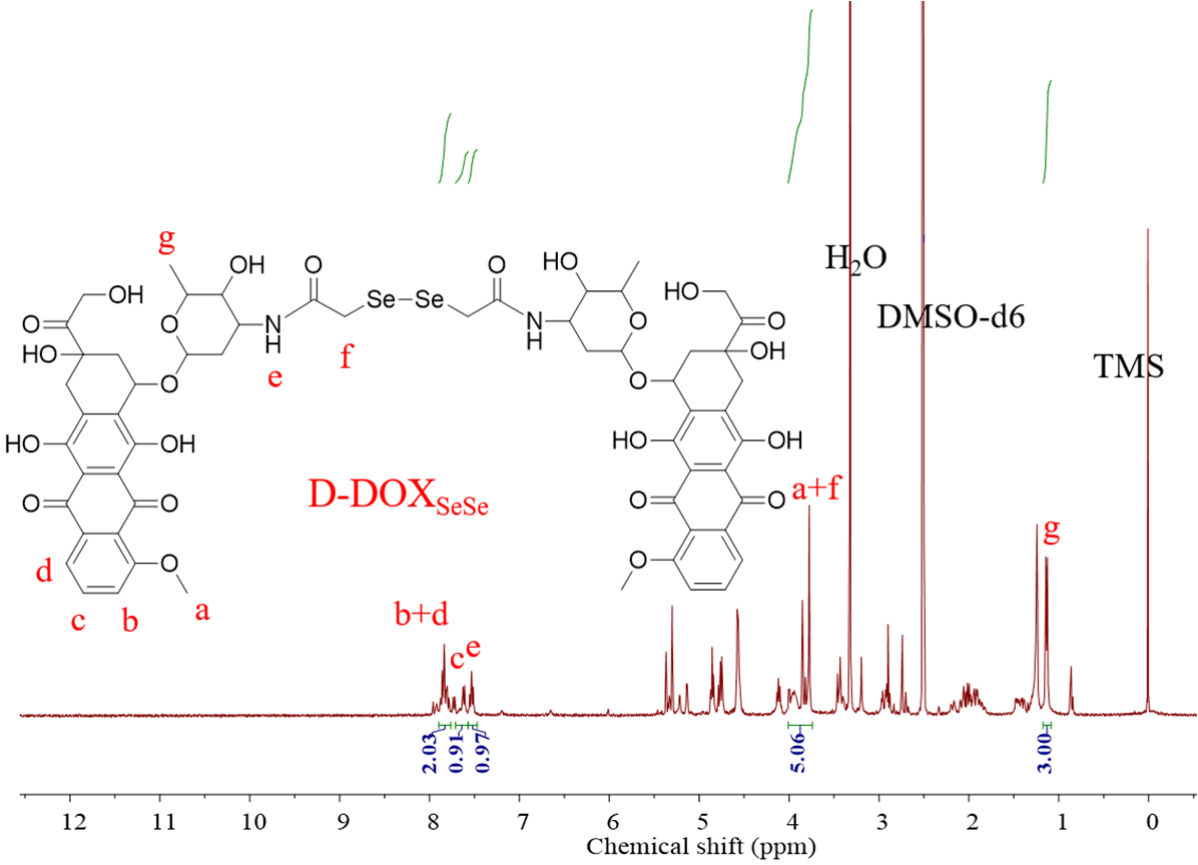
^1^H NMR spectrum of D-DOX_SeSe_.

In its ^1^H NMR spectrum (Figure 2), the proton in the amide group (H*e*) could be seen at δ = 7.51 − 7.41 ppm, revealing successful amidation. The proton signals on the benzene ring and the methyl group in DOX appeared at δ = 7.84 − 7.71 ppm (H*b + d*), δ = 7.65 − 7.54 ppm (H*c*), and δ = 1.15 − 1.04 ppm (H*g*). The integral area ratio between the protons on the methyl group and the benzene ring on DOX and the formed amide group was calculated as 3.00:2.94:0.97, near its theoretical value of 3:3:1, demonstrating the successful synthesis of the dimeric prodrug via the β-amide conjugation, as illustrated in Scheme 1. The proton signal of the -CH_2_- between the amide and diselenide (H*f*) bonds overlapped with those of the -OCH_3_ in DOX (H*a*) at δ = 3.96 − 3.70 ppm [32], which could not be used for molecular structure identification directly. However, compared with the ratio between the protons of benzene ring in DOX (H*b + d* at δ = 7.84 − 7.71 and H*c* at δ = 7.65 − 7.54) and H*a* (δ = 3.96 − 3.70 ppm) at 3:3, the integral area ratio between the signals was calculated as 5.06:2.94 in the product, also indicating the successful synthesis of the dimeric prodrug D-DOX_SeSe_, as illustrated in Scheme 1.

The proposed dimeric prodrug exhibited identical UV–Vis absorption in its dimethylsulfoxide (DMSO) solution as that of DOX solution at the same equivalent DOX molar concentration, meaning that dimerization did not affect its UV–Vis absorption. So, the DOX content of the D-DOX_SeSe_ could be determined by measuring the absorption of its DMSO solution at 480 nm with the UV–Vis technique and calculating it with the calibration curve of DOX in the DMSO solution. The DOX content of the D-DOX_SeSe_ was calculated as 1.54 × 10^−3^ mmol/g, very near to the theoretical value of 1.51 × 10^−3^ mmol/g. This result also demonstrated the successful synthesis of D-DOX_SeSe_.

### 2.2. The Fabrication and Characterization of Diselenide-Bridged Dimeric Prodrug Nanoparticles

**Figure 3:**
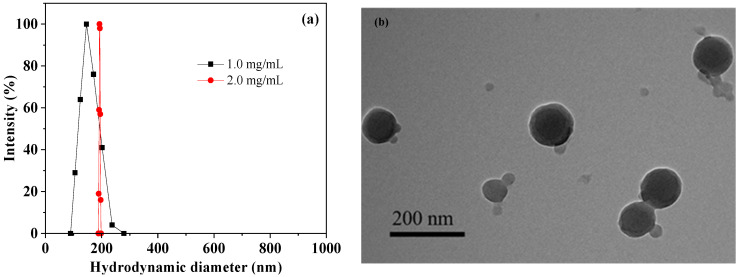
Hydrodynamic diameters of D-DOX_SeSe_ nanoparticles fabricated at different concentrations (**a**) and TEM image of D-DOX_SeSe_ nanoparticles fabricated at 1.0 mg/mL (**b**).

The D-DOX_SeSe_ nanoparticles were fabricated by dialyzing its DMSO solution at different concentrations against water (molecular weight cutoff (MWCO) of 1000). The hydrodynamic diameter of the D-DOX_SeSe_ nanoparticles increased with an increase in its concentration in the DMSO solution (Figure 3a). During the dialysis, the D-DOX_SeSe_ solution in a good solvent (DMSO) slowly transformed into a bad solvent (H_2_O), leading to the self-assembly of the D-DOX_SeSe_ via π–π stacking, hydrogen bonding, and hydrophobic interaction. Aiming to provide more efficient passive targeting via the EPR effect with a smaller diameter, the dimeric prodrug nanoparticles fabricated at 1.0 mg/mL were selected for further investigation.

The optimized D-DOX_SeSe_ nanoparticles exhibited a nearly spherical shape with a mean particle size of approximately 150 nm in the transmission electron microscopy (TEM) observation (Figure 3b). The value was near the mean hydrodynamic diameter of 155 nm from the dynamic light scattering (DLS) analysis, because they could hardly be swollen in an aqueous system due to high hydrophobicity.

### 2.3. Redox-Triggered Drug Release from Diselenide-Bridged Dimeric Prodrug Nanoparticles

**Figure 4:**
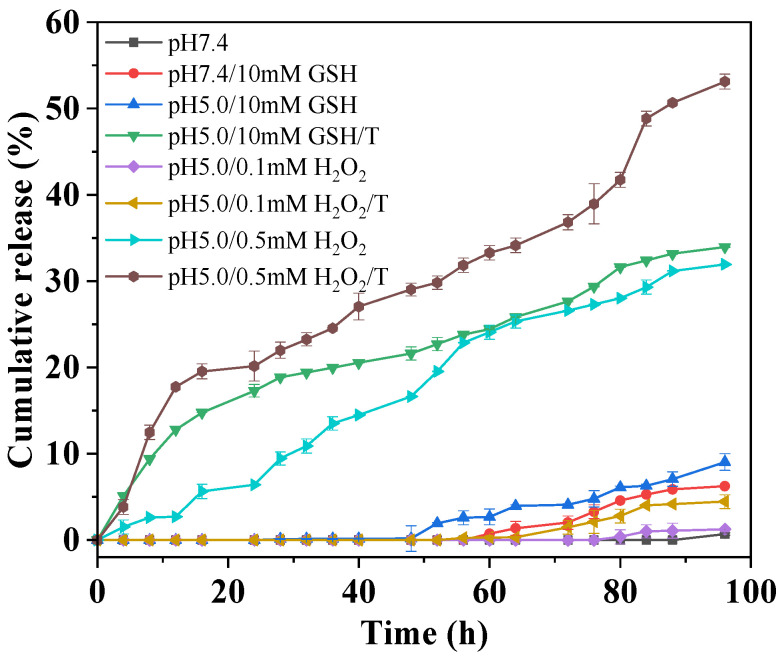
Redox-triggered drug release from D-DOX_SeSe_ nanoparticles in different media.

The redox-triggered drug release from the proposed D-DOX_SeSe_ nanoparticles was evaluated in different releasing media, with pH 7.4 phosphate-buffered saline (PBS) and pH 5.0 acetate-buffered solution (ABS) mimicking the acidity of the blood and the intracellular microenvironment with or without different GSH or H_2_O_2_ levels. As shown in Figure 4, there was no obvious drug release within the first 48 h in the acidic-releasing media with higher GSH or H_2_O_2_ levels, mimicking the tumor intracellular microenvironment (pH 5.0/10 mM GSH or pH5.0/0.1 mM H_2_O_2_). This demonstrates the safety of the proposed dimeric prodrug-based DSDS, avoiding both the drug leakage in the extracellular media and the mis-release in normal cells with lower GSH or H_2_O_2_ levels. After 96 h, the cumulative release was only <9% and <2%, respectively. The results indicated that the proposed D-DOX_SeSe_ nanoparticles could hardly release the drug in the in vitro-simulated tumor intracellular microenvironment. Increasing the H_2_O_2_ concentration to 0.5 mM, a cumulative release of 32% was achieved, due to the higher solubility of the released drug (seleninic acid (DOX-SeOOH)) owing to its higher polarity and hydrophilicity.

However, much higher cumulative release was obtained in such releasing media in the presence of surfactant (Tween-80 (T), 0.1%), indicating that the drug release was controlled by the solubility of the released drug [33,34], which was not the parent drug DOX but its derivates. It has been well reported that the diselenide bond possesses a higher redox-responsive sensitivity and can be cleaved with high GSH and ROS levels by transforming to selenol and seleninic acid [7], respectively. However, the aqueous solubility of such derivates was much lower than the parent drug DOX, due to their derivation from the amino group in DOX, which could be protonated and could enhance its aqueous solubility in the acidic media.

**Figure 5:**
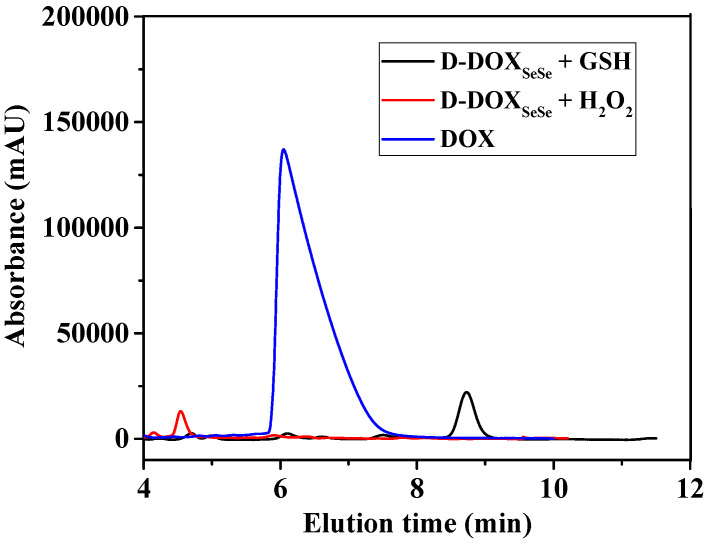
HPLC results of the D-DOX_SeSe_ nanoparticles after treatment with GSH and H_2_O_2_.

**Scheme 2:**
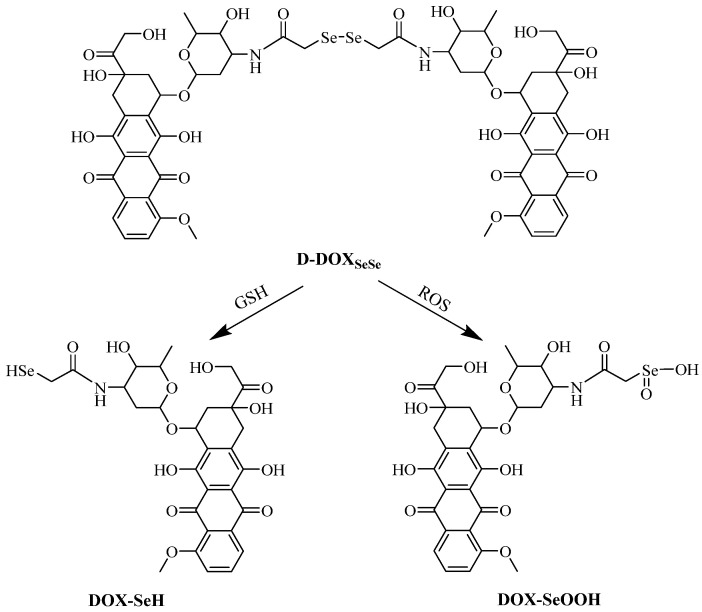
Reduction and oxidation-triggered degradation and drug release.

To further reveal the cleavage of the diselenide bond in the proposed dimeric prodrug, the D-DOX_SeSe_ nanoparticles were treated with 10 mM GSH or 0.5 mM H_2_O_2_ in methanol–water (vol: 3/7) at 37 °C for 24 h. The solution was analyzed by the HPLC technique with a flow phase of acetonitrile–water (vol: 3/7) containing 0.1% acetic acid, after filtration. A new signal emerged at 8.8 min after treating with GSH and 4.6 min after treating with H_2_O_2_ (Figure 5), indicating that the derivatives selenol (DOX-SeH) and seleninic acid (DOX-SeOOH) were released after cleaving with GSH and H_2_O_2_, respectively (Scheme 2).

Due to amidation, the D-DOX_SeSe_ became more hydrophobic than DOX. Furthermore, it formed compact self-assemblers via π–π stacking, hydrogen bonding, and hydrophobic interaction, which restricted the diffusion of the stimuli into the D-DOX_SeSe_ nanoparticles. Thus, only the diselenide bond on the surface of the D-DOX_SeSe_ nanoparticles could be cleaved. However, triggered by the reduction of 10 mM GSH, the selenol derivative (DOX-SeH) was produced. It exhibited lower aqueous solubility but could be adsorbed onto the surface of the D-DOX_SeSe_ nanoparticles via the above weak interactions, shielding against the attacking of GSH on the dimeric prodrug. As a result, no obvious drug release could be achieved. However, with the help of the solubilization effect of Tween-80, the produced selenol derivative (DOX-SeH) could be emulsified into the releasing media, facilitating the exposure and reduction-triggered cleavage of the dimeric prodrug molecules. Thus, a sustained drug release was obtained. Although the dimeric prodrug might also be emulsified with Tween-80 as the selenol derivative (DOX-SeH), it could not penetrate through the dialysis bag (MWCO of 1000) due to its higher molecular weight.

Distinct from the reduction-triggered drug release with a high GSH level, the cumulative release was also negligible in the releasing medium of pH5.0/0.1 mM H_2_O_2_ even with 0.1% Tween-80. This demonstrated that the diselenide bond could easily be broken by the reduction of 10 mM GSH, while it was stable with the oxidation of 0.1 mM H_2_O_2_ in the simulated tumor intracellular microenvironment. However, the intracellular ROS level could be upregulated because of the consumption of GSH during the reduction-triggered cleavage of the diselenide-bridged doxorubicin dimeric prodrug, accelerating the oxidation-triggered drug release in the real tumor intracellular microenvironment.

### 2.4. Cellular Uptake and Tumor-Selective Cytotoxicity of Diselenide-Bridged Dimeric Prodrug Nanoparticles

**Figure 6:**
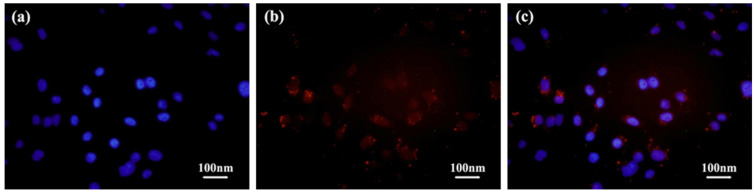
CLSM images of HepG2 cells after incubation with the D-DOX_SeSe_ nanoparticles (15 μg/mL) for 24 h: (**a**) nuclei stained with DAPI, (**b**) DOX, and (**c**) the merged image.

After co-incubation with the D-DOX_SeSe_ nanoparticles (15 μg/mL) for 24 h, a human liver cancer cell line (HepG2 hepatocellular carcinoma) was analyzed with confocal laser scanning microscopy. As shown in Figure 6, the red fluorescence of DOX could be seen in both the cytoplasm and the nuclei, which were stained with 4, 6-diaminyl-2-phenylindole dihydrochloride (DAPI) and emitted blue fluorescence. The results indicated that the D-DOX_SeSe_ nanoparticles could be internalized into the HepG2 cells, and they released the DOX derivatives in the cytoplasm with the GSH/ROS dual-triggering.

**Figure 7:**
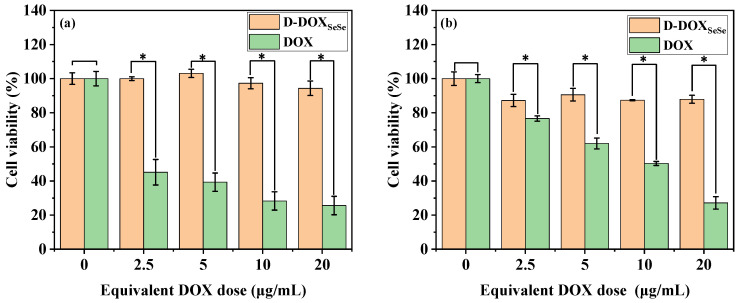
Cell viability assay in L02 (**a**) and HepG2 (**b**) cells of D-DOX_SeSe_ nanoparticles and free DOX with different concentrations for 48 h, respectively. Values are expressed as mean ± SD (n = 6); * denotes significant difference *p* < 0.05.

Finally, the tumor-selective toxicity of the D-DOX_SeSe_ nanoparticles on the HepG2 cells and human normal liver cell line (L02 hepatocytes) was assessed in vitro with MTT assays, in comparison with free DOX. For the human normal liver cell line, free DOX showed dose-dependent cytotoxicity, while the D-DOX_SeSe_ nanoparticles did not show obvious cytotoxicity with a cell viability rate of >95% at an equivalent DOX dose of 20 μg/mL (Figure 7a). As for the human liver cancer cell line, both systems showed dose-dependent cytotoxicity (Figure 7b). The cell viability was higher after incubation with the D-DOX_SeSe_ nanoparticles in comparison with free DOX at the same DOX dose due to the slow release of derivatives selenol (DOX-SeH) and seleninic acid (DOX-SeOOH) in the tumor cells in which the GSH and ROS levels were higher than those in normal cells. Furthermore, the derivatization from the amino group in DOX decreased its antitumor efficacy by declining the insertion in DNA [35]. Therefore, the proposed D-DOX_SeSe_ nanoparticles are expected to serve as a long-acting sustained-release system to eradicate any residual or latent cancerous cells, which might lead to the recurrence of tumors [36].

Generally, the antitumor efficacy of DOX derivatives is usually determined by their solubility, which controls their release and their insertion in DNA. Although the diselenide bond could hardly be cleaved in the presence of ROS in tumor cells, the reduction-triggered cleavage would cause GSH consumption, therefore disrupting the redox balance and upregulating the ROS level in the tumor cells. An accelerated oxidation-triggered drug release was expected by exogenous irradiation [20,21,22,23,24,25], which could produce more ROS. Thus, a personalized chemotherapy regimen can be proposed with a fast drug release in the early stage with the help of exogenous irradiation and a slow sustained drug release in the late stage to eradicate any residual or latent cancerous cells.

## 3. Discussion

The proposed diselenide-bridged doxorubicin dimeric prodrug (D-DOX_SeSe_) was revealed to be stable in both the extracellular environment and the intracellular microenvironment in normal cells but was triggered to release DOX-SeH in the tumor intracellular microenvironment, which was controlled by its solubility. Although the selenide bond has higher redox-responsive sensitivity than the disulfide bond, the oxidation-triggered cleavage of the diselenide bond needs a much higher H_2_O_2_ level than in the tumor intracellular microenvironment, especially when diselenide bonds are combined with α-amide, as revealed in the present work. A slow solubility-controlled drug release was achieved with the proposed diselenide-bridged dimeric prodrug, indicating a promising application as a safe long-acting sustained-release system in tumor treatment.

However, it was found that the α-amide derivatives selenol (DOX-SeH) and seleninic acid (DOX-SeOOH) could hardly transform into the parent drug DOX by the hydrolysis of the amide group induced by the selenol and seleninic acid groups, although the selenol and seleninic acid groups have been revealed to induce the hydrolysis of α-ester [30], γ-carbonate, and γ-carbamate groups [30,37,38,39,40]. On the other hand, the thiol group has also been reported to induce the acid-promoted hydrolysis of α-amide conjugation for the redox-triggered DOX release from the polymer prodrug conjugated with thiodiacetic acid [31].

This may be due to the lower electronegativity of the selenide atom, which endows a higher level of redox-responsive sensitivity for the cleavage of the diselenide bond but a lower inducing effect for the hydrolysis of the released drug derivatives. It could only induce the hydrolysis of α-ester, γ-carbonate, and γ-carbamate groups with lower hydrolysis stability but could not induce the hydrolysis of the α-amide group with higher hydrolysis stability owing to a “partial” π-bond between nitrogen and carbonyl carbon. A combination of conjugations should be considered in designing a self-immolation traceless conjugation in prodrugs, besides the length of diselenide bond-containing linkages [41]. Such understanding is helpful in the future design of diselenide-containing prodrugs for better tumor-specific chemotherapy.

## 4. Materials and Methods

### 4.1. Materials and Reagents

Selenium (Se, ≥99.99%), chloroacetic acid (ClCH_2_COOH, 99%), and glutathione (GSH, 97%) were purchased from Shanghai Aladdin Bio-Chem Technology Co., Ltd. (Shanghai, China). Sodium borohydride (NaBH_4_, 96%) was purchased from Sinopharm Chemical Reagent Co., Ltd. (Shanghai, China). Hydrogen peroxide (H_2_O_2_, 30%) and sodium carbonate (Na_2_CO_3_,99.5%) were purchased from Damao Chemical Reagent Factory (Tianjin, China). 1-(3-dimethylaminopropyl)-3-ethylcarbodiimide hydrochloride (EDC·HCl, 99%) and N-hydroxysuccinimide (NHS, 98%) were provided from JK Scientific, Ltd. (San Jose, CA, USA). Doxorubicin hydrochloride (DOX·HCl, 99.4%) was bought from Beijing Huafeng United Technology Co., Ltd. (Beijing, China). All other reagents and solvents were of analytical grade and used directly as received. Deionized water was used throughout the experiments.

### 4.2. Analysis and Characterization

The molecular analysis of DSeDAA and the proposed diselenide-bridged doxorubicin dimeric prodrug (D-DOX_SeSe_) was conducted with 400 MHz ^1^H NMR (JNM-ECS 400 M, JEOL, Tokyo, Japan) in DMSO-d6. C, N, and H elemental analyses were performed on an Elementar vario EL instrument. The reduction and oxidation-triggered cleavage of the diselenide-bridged dimeric prodrug were studied on a Waters 2690D HPLC system, equipped with a Waters model 510 HPLC pump (Waters, Milford, MA, USA), a UV–Vis detector, and a Symmetry C18 column. The morphology and particle size of the D-DOX_SeSe_ nanoparticles were observed with transmission electron microscopy (TEM, JEM-2100, Tokyo, Japan) sampling with aqueous dispersion. The hydrodynamic diameter and distribution of the D-DOX_SeSe_ nanoparticles were measured using dynamic light scattering (DLS, BI-200SM, Brookhaven, NY, USA) in aqueous dispersion. A TU-1901 UV–Vis spectrometer (Beijing Purkinje General Instrument Co., Ltd., Beijing, China) was used to measure the DOX concentrations in different media at 480 nm, which were calculated with the calibration curves of DOX in the different media as follows: DMSO: absorbance = 10,400 × concentration (mmol/mL) − 0.00183 (R^2^ = 0.9984); pH 7.4 PBS: absorbance = 10,357 × concentration (mmol/mL) + 0.00427 (R^2^ = 0.9968); pH 5.0 ABS: absorbance = 12,132 × concentration (mmol/mL) + 0.00367 (R^2^ = 0.9980); pH 7.4 PBS + 10 mM GSH: absorbance = 11,416 × concentration (mmol/mL) + 0.00500 (R^2^ = 0.9947); pH 5.0 ABS + 10 mM GSH: absorbance = 12,283 × concentration (mmol/mL) + 0.00490 (R^2^ = 0.9963); pH 5.0 ABS + 0.1 mM H_2_O_2_: absorbance = 11,648 × concentration (mmol/mL) + 0.00428 (R^2^ = 0.9971); pH 5.0 ABS + 0.5 mM H_2_O_2_: absorbance = 11,356 × concentration (mmol/mL) + 0.00273 (R^2^ = 0.9987).

### 4.3. Synthesis Procedure

Diselenodiacetic acid (DSeDAA) was synthesized according to the reported procedure with minor modifications [31]. Typically, Se (1.6 g, 0.02 mol, 1.0 eq) was dispersed in 20 mL of water in an ice-water bath under an Ar atmosphere. A NaBH_4_ (1.5 g, 0.04 mol, 2.0 eq) aqueous solution was added to the Se dispersion. After the solution became colorless, Se (1.6 g, 0.02 mol, 1.0 eq) was added, and the mixture was stirred at 60 °C until the Se powder was completely dissolved. Then, 20 mL of the chloroacetic acid aqueous solution (3.8 g, 0.04 mol, 2.0 eq) at pH 9~10, which was adjusted with the Na_2_CO_3_ saturated solution, was added, and the reaction was conducted for 6 h. After cooling to room temperature, the resultant solution was adjusted to pH 3~4 and extracted with ethyl acetate. After drying with anhydrous Na_2_SO_4_, the crude product was obtained by vaporization under reduced vacuum. Finally, DSeDAA was obtained as yellowish crystals with a yield of 35.8%, after recrystallization in ethyl acetate/*n*-hexane (*v*/*v* of 1:1).

DOX·HCl (100.0 mg, 0.172 mmol, 2.0 eq) and triethylamine (30 μL, 2.4 eq) were dissolved in 20 mL DMF for 1 h to produce free DOX. After DSeDAA (24.0 mg, 0.086 mmol, 1.0 eq), EDC·HCl (50.0 mg, 0.259 mmol 3.0 eq), and NHS (30.0 mg, 0.259 mmol, 3.0 eq) were added, the reaction was conducted by stirring for 24 h at room temperature in the dark. The proposed diselenide-bridged doxorubicin dimeric prodrug (D-DOX_SeSe_) was purified by dialyzing the resultant solution with DMF for a day and then with water for 3 days (MWCO of 1000) by changing the dialysate every 8 h, collected by centrifugation (10,000 rpm, 5 min) and finally dried in vacuum at 40 °C (Yield: 61%). Its purity was revealed with TLC on a silica gel plate.

### 4.4. Redox-Triggered Drug Release

The D-DOX_SeSe_ nanoparticles (1.0 mg) were dispersed in 10 mL of release media with different pH values, GSH levels, and H_2_O_2_ levels with or without 0.1% Tween-80. The dispersion was dialyzed (MWCO = 1000) in 100 mL of the corresponding releasing medium in an IS-RSD3 incubation shaker at 37 °C. At certain time intervals, 5.0 mL of the dialysate was taken out to measure the DOX concentration on the UV–Vis spectrometer at 480 nm, and 5.0 mL of the fresh buffer solution was added to maintain a constant volume.

### 4.5. Redox-Triggered Drug Release

The DOX content in the proposed diselenide-bridged doxorubicin dimeric prodrug (D-DOX_SeSe_) was determined by measuring the UV–Vis absorption of its DMSO solution at 480 nm on a TU-1901 UV–Vis spectrometer and calculated with the calibration curve of DOX in the DMSO solution.

### 4.6. In Vitro Cellular Uptake and Cytotoxicity

The L02 and HepG2 cells were incubated in a 96-well plate with a concentration of 1 × 10^5^ per well at 37 °C for 48 h.

For cellular uptake, the HepG2 cells were incubated with 15 μg/mL of D-DOX_SeSe_ nanoparticles for 24 h. After being fixed with paraformaldehyde solution, washed twice using PBS, stained with DAPI, and then washed twice using PBS, the HepG2 cells were analyzed with an inverted fluorescence microscope (OLYMPUS, IX71) (DAPI at 405 nm and DOX at 480 nm).

For cytotoxicity, different concentrations of the D-DOX_SeSe_ nanoparticles or free DOX were added to perform co-incubation for 48 h. The cell viability was assessed with MTT assays, using the Enzyme-Linked Immunosorbent Assay Appliance at 490 nm.

## 5. Conclusions

In summary, a diselenide-bridged doxorubicin dimeric prodrug (D-DOX_SeSe_) was designed as a redox-triggered drug self-delivery system (DSDS) for tumor-specific chemotherapy. By combining with the α-amide group, it showed redox-triggered cleavage at higher GSH and ROS levels but only a slow solubility-controlled reduction-triggered drug release in the in vitro drug release profiles in the simulated tumor intracellular microenvironment, holding promise as a safe long-acting GSH-triggered slow sustained-release system in tumor treatment, as revealed by the in vitro cellular experiment results.

By comparing with the reported works on the redox-responsive disulfide or diselenide-containing prodrugs, the interaction between the redox-responsive conjugation and neighboring ester or amide groups was also explored, which was suggested as an important concern in the future design of self-immolation traceless conjugations in prodrugs. Despite the higher redox-responsive sensitivity of the diselenide bond compared to the disulfide bond, which facilitates its cleavage with the reduction of GSH or oxidation with ROS, it was concluded that a self-immolation traceless conjugation could be designed by integrating the disulfide bond with the ester or amide group or the diselenide bond with the ester group, but not the combination of the diselenide bond and the α-amide group due to the double-edged sword effect of the selenide atom with lower electronegativity in comparison with the sulfur atom. This finding has not been reported in previous works; thus, this study presents a theoretical foundation for precise tumor-specific chemotherapy.

## Data Availability

The data presented in this study are available in this article.

## References

[1] Kalyane D., Raval N., Maheshwari R., Tambe V., Kalia K., Tekade R.K. (2019). Employment of enhanced permeability and retention effect (EPR): Nanoparticle-based precision tools for targeting of therapeutic and diagnostic agent in cancer. Mater. Sci. Eng. C.

[2] Wang D.-D., Zhang X.-N. (2021). Advances in receptor modulation strategies for flexible, efficient, and enhanced antitumor efficacy. J. Control. Release.

[3] Sun T., Jiang C. (2023). Stimuli-responsive drug delivery systems triggered by intracellular or subcellular microenvironments. Adv. Drug Deliv. Rev..

[4] Mirhadi E., Mashreghi M., Maleki M.F., Alavizadeh S.H., Arabi L., Badiee A., Jaafari M.R. (2020). Redox-sensitive nanoscale drug delivery systems for cancer treatment. Int. J. Pharm..

[5] Álvarez-Pérez M., Ali W., Marć M.A., Handzlik J., Domínguez-Álvarez E. (2018). Selenides and Diselenides: A Review of Their Anticancer and Chemopreventive Activity. Molecules.

[6] Miao Q., Xu J.Y., Lin A.J., Wu X.M., Wu L., Xie W.J. (2018). Recent Advances for the Synthesis of Selenium-containing Small Molecules as Potent Antitumor Agents. Curr. Med. Chem..

[7] Shi Z.F., Liu J.F., Tian L., Li J.Y., Gao Y., Xing Y., Yan W.J., Hua C.Y., Xie X.L., Liu C. (2022). Insights into stimuli-responsive diselenide bonds utilized in drug delivery systems for cancer therapy. Biomed. Pharmacother..

[8] Mollazadeh S., Mackiewicz M., Yazdimamaghani M. (2022). Recent advances in the redox-responsive drug delivery nanoplatforms: A chemical structure and physical property perspective. Mater. Sci. Eng. C.

[9] Abed H.F., Abuwatfa W.H., Husseini G.A. (2022). Redox-Responsive Drug Delivery Systems: A Chemical Perspective. Nanomaterials.

[10] Yadav S., Ramesh K., Reddy O.S., Karthika V., Kumar P., Jo S.H., Yoo S.I., Park S.H., Lim K.T. (2023). Redox-Responsive Comparison of Diselenide and Disulfide Core-Cross-Linked Micelles for Drug Delivery Application. Pharmaceutics.

[11] Wang J., Liu J., Lu D.Q., Chen L.J., Yang R.J., Liu D.H., Zhang B. (2022). Diselenide-crosslinked carboxymethyl chitosan nanoparticles for doxorubicin delivery: Preparation and in vivo evaluation. Crabohydr. Polym..

[12] Kim S.G., Robby A.I., Lee B.C., Lee G., Park S.Y. (2021). Mitochondria-targeted ROS- and GSH-responsive diselenide-crosslinked polymer dots for programmable paclitaxel release. J. Ind. Eng. Chem..

[13] Birhan Y.S., Darge H.F., Hanurry E.Y., Andrgie A.T., Mekonnen T.W., Chou H.Y., Lai J.Y., Tsai H.C. (2020). Fabrication of Core Crosslinked Polymeric Micelles as Nanocarriers for Doxorubicin Delivery: Self-Assembly, In Situ Diselenide Metathesis and Redox-Responsive Drug Release. Pharmaceutics.

[14] Tian Y.F., Lei M., Yan L.K., An F.F. (2020). Diselenide-crosslinked zwitterionic nanogels with dual redox-labile properties for controlled drug release. Polym. Chem..

[15] Li M.X., Li Q.Y., Hou W., Zhang J.N., Ye H.M., Li H.A., Zeng D.P., Bai J. (2020). A redox-sensitive core-crosslinked nanosystem combined with ultrasound for enhanced deep penetration of nanodiamonds into tumors. RSC Adv..

[16] Lee H.L., Hwang S.C., Nah J.W., Kim J., Cha B., Kang D.H., Jeong Y.I. (2018). Redox- and pH-Responsive Nanoparticles Release Piperlongumine in a Stimuli-Sensitive Manner to Inhibit Pulmonary Metastasis of Colorectal Carcinoma Cells. J. Pharm. Sci..

[17] Zhai S.D., Hu X.L., Hu Y.J., Wu B.Y., Xing D. (2017). Visible light-induced crosslinking and physiological stabilization of diselenide-rich nanoparticles for redox-responsive drug release and combination chemotherapy. Biomaterials.

[18] Deepagan V.G., Kwon S., You D.G., Nguyen V.Q., Um W., Ko H., Lee H., Jo D.G., Kang Y.M., Park J.H. (2016). In situ diselenide-crosslinked polymeric micelles for ROS-mediated anticancer drug delivery. Biomaterials.

[19] Li C.T., Huang W., Zhou L.Z., Huang P., Pang Y., Zhu X.Y., Yan D.Y. (2015). PEGylated poly(diselenide-phosphate) nanogel as efficient self-delivery nanomedicine for cancer therapy. Polym. Chem..

[20] Wang J., Xu W.G., Zhang N., Yang C.S., Xu H.W., Wang Z.T., Li B.S., Ding J.X., Chen X.S. (2021). X-ray-responsive polypeptide nanogel for concurrent chemoradiotherapy. J. Control. Release.

[21] Shao D., Zhang F., Chen F.M., Zheng X., Hu H.Z., Yang C., Tu Z.X., Wang Z., Chang Z.M., Lu J.N. (2020). Biomimetic Diselenide-Bridged Mesoporous Organosilica Nanoparticles as an X-ray-Responsive Biodegradable Carrier for Chemo-Immunotherapy. Adv. Mater..

[22] Zhang L.X., Zhang S.T., Xu J.Y., Li Y.Y., He J.L., Yang Y., Huynh T., Ni P.H., Duan G.X., Yang Z.X. (2020). Low-Dose X-ray-Responsive Diselenide Nanocarriers for Effective Delivery of Anticancer Agents. ACS Appl. Mater. Interfaces.

[23] Cao W., Zhang X., Miao X., Yang Z., Xu H. (2013). gamma-Ray-responsive supramolecular hydrogel based on a diselenide-containing polymer and a peptide. Angew. Chem. Int. Ed..

[24] Rizwan A., Gulfam M., Jo S.-H., Seo J.-W., Ali I., Vu T.T., Joo S.-B., Park S.-H., Lim K.T. (2023). Gelatin-based NIR and reduction-responsive injectable hydrogels cross-linked through IEDDA click chemistry for drug delivery application. Eur. Polym. J..

[25] Zhu R., He Q., Li Z.L., Ren Y.H., Liao Y.X., Zhang Z.J., Dai Q., Wan C.Y., Long S.H., Kong L.Y. (2022). ROS-cleavable diselenide nanomedicine for NIR-controlled drug release and on-demand synergistic chemo-photodynamic therapy. Acta Biomater..

[26] Wei D., Yu Y., Zhang X., Wang Y., Chen H., Zhao Y., Wang F., Rong G., Wang W., Kang X. (2020). Breaking the intracellular redox balance with diselenium nanoparticles for maximizing chemotherapy efficacy on patient-derived xenograft models. ACS Nano.

[27] Deng K.L., Tian H.L., Zhang T.T., Gao Y.J., Nice E.C., Huang C.H., Xie N., Ye G.L., Zhou Y.P. (2024). Chemo-photothermal nanoplatform with diselenide as the key for ferroptosis in colorectal cancer. J. Control. Release.

[28] Li S.M., Shan X.Z., Wang Y.Q., Chen Q., Sun J., He Z.G., Sun B.J., Luo C. (2020). Dimeric prodrug-based nanomedicines for cancer therapy. J. Control. Release.

[29] Zhang H.J., Pan J.X., Wang T.T., Lai Y., Liu X.Y., Chen F.M., Xu L.M., Qu X.W., Hu X.L., Yu H.J. (2022). Sequentially Activatable Polypeptide Nanoparticles for Combinatory Photodynamic Chemotherapy of Breast Cancer. ACS Appl. Mater. Interfaces.

[30] Lu S.J., Xia R., Wang J., Pei Q., Xie Z.G., Jing X.B. (2021). Engineering paclitaxel prodrug nanoparticles via redox-activatable linkage and effective carriers for enhanced chemotherapy. ACS Appl. Mater. Interfaces.

[31] Zuo S.Y., Sun B.J., Yang Y.X., Zhou S., Zhang Y., Guo M.R., Sun M.C., Luo C., He Z.G., Sun J. (2020). Probing the Superiority of Diselenium Bond on Docetaxel Dimeric Prodrug Nanoassemblies: Small Roles Taking Big Responsibilities. Small.

[32] Hao J.X., Wang J.L., Pan H., Sang Y.L., Wang D.Z., Wang Z.Y., Ai J., Lin B., Chen L.J. (2022). pH-redox responsive polymer-doxorubicin prodrug micelles studied by molecular dynamics, dissipative particle dynamics simulations and experiments. J. Drug Deliv. Sci. Technol..

[33] Liu P. (2023). Polyprodrugs for tumor chemotherapy: From molecular structure to drug release performance. J. Mater. Chem. B.

[34] Li X.M., Liu P. (2022). Synthesis and self-assembly of an acid/reduction co-triggered degradable amphiphilic copolyprodrug as a tumor-selective drug self-delivery system. J. Mater. Chem. B.

[35] Song Q., Wang X., Wang Y.Q., Liang Y.Q., Zhou Y.X., Song X.N., He B., Zhang H., Dai W.B., Wang X.Q. (2016). Reduction Responsive Self-Assembled Nanoparticles Based on Disulfide-Linked Drug–Drug Conjugate with High Drug Loading and Antitumor Efficacy. Mol. Pharm..

[36] Wolinsky J.B., Colson Y.L., Grinstaff M.W. (2012). Local drug delivery strategies for cancer treatment: Gels, nanoparticles, polymeric films, rods, and wafers. J. Control. Release.

[37] He X., Zhang J.X., Li C., Zhang Y., Lu Y.F., Zhang Y.J., Liu L.S., Ruan C.H., Chen Q.J., Chen X.L. (2018). Enhanced bioreduction-responsive diselenide-based dimeric prodrug nanoparticles for triple negative breast cancer therapy. Theranostics.

[38] Zhao J.T., Wang Z.H., Zhong M., Xu Q.H., Li X.M., Chang B.B., Fang J.G. (2021). Integration of a Diselenide Unit Generates Fluorogenic Camptothecin Prodrugs with Improved Cytotoxicity to Cancer Cells. J. Med. Chem..

[39] Chen M., Zhang M., Lu X., Li Y.F., Lu C. (2023). Diselenium-linked dimeric prodrug nanomedicine breaking the intracellular redox balance for triple-negative breast cancer targeted therapy. Eur. J. Pharm. Biopharm..

[40] Sun B.J., Luo C., Zhang X.B., Guo M.C., Sun M.C., Yu H., Chen Q., Yang W.Q., Wang M.L., Zuo S.Y. (2019). Probing the impact of sulfur/selenium/carbon linkages on prodrug nanoassemblies for cancer therapy. Nat. Commun..

[41] Li L.X., Zuo S.Y., Dong F.D., Liu T., Gao Y.L., Yang Y.X., Wang X., Sun J., Sun B.J., He Z.G. (2021). Small changes in the length of diselenide bond-containing linkages exert great influences on the antitumor activity of docetaxel homodimeric prodrug nanoassemblies. Asia J. Pharm. Sci..

